# SOCIAL INTERACTIONS OF COMMUNITY SPACE ATTENDEES AND ANTICIPATED FUNCTION OF THE SPACE AMID THE COVID-19 PANDEMIC

**DOI:** 10.1093/geroni/igac059.2001

**Published:** 2022-12-20

**Authors:** Mai Takase, Ryogo Ogino, Ryoichi Nitanai, Riko Nakayama, Hongjik Kim, Neo Kazembe, Jun Goto, Katsuya Iijima

**Affiliations:** The University of Tokyo, Tokyo, Tokyo, Japan; Saga University, Saga, Saga, Japan; The University of Tokyo, Tokyo, Tokyo, Japan; The University of Tokyo, Tokyo, Tokyo, Japan; The University of Tokyo, Tokyo, Tokyo, Japan; The University of Tokyo, Tokyo, Tokyo, Japan; Tokai University, Hiratsuka, Tokyo, Japan; The University of Tokyo, Tokyo, Tokyo, Japan

## Abstract

**Introduction:**

A regional community space in Japan, Chiiki-Katsudokan, was founded to facilitate the social interactions of older adults through activities. Meanwhile, the COVID-19 pandemic occasionally forced this space to close or limited the volume of its participants. In this research, the operation of the space was reviewed by monitoring the changes in the social interactions of the attendees, and by investigating the anticipated function from the attendees' needs. Method: A semi-structured interview targeting the attendees of Chiiki-Katsudokan was conducted in December 2021 (N=19, main age:80s). The level of social interaction before and during the pandemic and the newly anticipated function of the space were examined.

**Results:**

First, deep and light interactions were observed. Those with deep interactions initially had wide social connections and used Chiiki-Katsudokan to interact with friends. Meanwhile, those with light interactions only talked to other attendees while attending the space. During the quarantine, those with deep interactions stayed connected with others and met privately, while those with light interactions faced a higher risk of social isolation. Second, the most popular newly anticipated functions of the space were "Place to gather with friends (n=14)" and "Interaction with younger generation (n=14)." "Opportunity to learn about the new pandemic-lifestyle (n=11)" was also rated high, while need for "online events" was rated the lowest (n=7).

**Conclusion:**

The pandemic re-emphasized older adults' need for direct interaction. Low interest in technology-based countermeasures suggested that community spaces should expand their operation method while considering offline methods (e.g., pen-pal system) that could enhance social interaction.

